# The effect of quarantine policy on pollution emission and the usage of private transportation in urban areas

**DOI:** 10.1038/s41598-024-66685-8

**Published:** 2024-07-08

**Authors:** Yihang Hong, Ke Lu

**Affiliations:** 1 School of Management Science and Engineering, Nanjing University of Information Science & Technology, Nanjing, 210044 China; 2 Department of Economics, University of Reading, White Knight, RG66UR UK

**Keywords:** Urban transportation, Machine learning algorithm, Time value theory, SARS-CoV-2, Black carbon aerosols, Environmental impact, Environmental economics, Sustainability

## Abstract

Governmental policies, regulations, and responses to the pandemic can benefit from a better understanding of people's resulting behaviours before, during, and after COVID-19. To avoid the inelasticity and subjectivity of survey datasets, several studies have already used some objective variables like air pollutants to estimate the potential impacts of COVID-19 on the urban transportation system. However, the usage of reactant gases and a narrow time scale might weaken the results somehow. Here, both the objective passenger volume of public transport and the concentration of private traffic emitted black carbon (BC) from 2018 to 2023 were collected/calculated to decipher the potential relationship between public and private traffic during the COVID-19 period. Our results indicated that the commuting patterns of citizens show significant (p < 0.01) different patterns before, during, and after the pandemic. To be specific, public transportation showed a significant (p < 0.01) positive correlation with private transportation before the pandemic. This public transportation was significantly (p < 0.01) affected by the outbreaks of COVID-19, showing a significant (p < 0.01) negative correlation with private transportation. Such impacts of the virus and governmental policy would affect the long-term behaviour of individuals and even affect public transportation usage after the pandemic. Our results also indicated that such behaviour was mainly linked to the governmental restriction policy and would soon be neglected after the cancellation of the restriction policy in China.

## Introduction

After the first announcement of the severe acute respiratory syndrome coronavirus 2 (SARS-CoV-2) outbreaks, the novel coronavirus dramatically swapped the world. Many places experienced outbreaks of the SARS-CoV-2 Alpha, known as the pandemic of coronavirus disease (COVID-19). A lot of temporary control restrictions were conducted by the local government, including remote working, closure of shops, transport system shutdowns, travel restrictions, social distancing, self-isolation, and even lockdown of the whole city^1–3^, to cut down the transmission of this novel coronavirus^4^. In the beginning, most Chinese local governments took the lockdown policy to handle the increasing SARS-CoV-2 cases. With the increase in vaccination^5^, the contacts infected ratio further declined, and then, some local governments chose the limited quarantine policy to reopen^6^. Then the cities could gradually restore the social and economic order until the SARS-CoV-2 Delta and Omicron outbreaks happened again.

The infected individuals of SARS-CoV-2 Omicron could pass it on to more close contacts (regardless of their vaccination status) than Delta, highlighting the high transmission ability of the Omicron variant^7^. Besides, the results of Kim, et al.^8^ indicated that the duration from the Delta epidemic period to Omicron was decreased from 135 to 35 days with a ~ 49% decrease in the total death, while the total confirmed cases increased by ~ 4 times. With the fatality rate further decreased (SARS-CoV-2 Alpha mutates into Delta and Omicron)^8–10^ and the transmission ability increased^11,12^, China experienced lots of outbreaks and lockdowns within the following one year until the publication of the “10 new measures” guideline to optimize COVID-19 response on 8th September 2022. Today, although the impacts become lower and lower, the COVID-19 pandemic still affects our daily lives to some extent^13–16^.

Many cities observed a significant decrease in both anthropogenic pollutants and individual mobilities during the first lockdown period^4,16–22^. Even after the pandemic, some previous research still observed some abnormal variations of both air pollutants^23^ and daily emission pattern^24^. During the lockdown period, people usually tried to reduce their mobility (i.e., online meetings and working) and keep social distance (i.e., using more private transportation) to reduce the risk of contagion during the COVID-19 period^1,25^. Such experiences and knowledge gained from the pandemic could significantly change the daily commuting choice of individuals even after the COVID-19 outbreaks^14^, especially the public transport^1,4^. Meanwhile, some other researchers have reported that the impacts of these novel coronaviruses would coexist with us in the future for a long time^13^ while others have not^14^, highlighting the urgency of understanding the severe pandemic impacts on the sociology systems.

To decipher the impacts of the virus on human activity, the collection of easily obtained anthropogenic-derived data with limited influencing factors was more and more essential. Previously, lots of research using reactive gases [i.e., carbon monoxide (CO), sulfur dioxide (SO_2_), nitrogen oxides (NO_x_), and methane (CH_4_)], carbon dioxide (CO_2_), or particle matter (PM) to estimate the variation of transportations^26^. However, since the reactive gases would experience lots of atmospheric reactions, CO_2_ concentration is usually dependent on the meteorological conditions, and PM contains lots of mixed secondary and natural emission sources, then, these air pollutants might not reflect the panoramic view of transportation emissions.

Black carbon (BC) aerosol is one kind of these anthropogenic linked components^27,28^, having been widely used in transportation research preciously^29–31^. Not like other reactive gases, BC was stable in the atmosphere and widely observed over the world^32,33^. Although BC still contains some biomass burning, factory, and residential emission sources, it could be used to reflect the traffic activities after some source apportionment techniques like stable or radiocarbon isotope (^13^C and ^14^C) analysis^34,35^, positive-definite matrix factorization (PMF)^36^, Aethalometer model^37^, chemical transportation model (CTM)^38^, and machine learning algorithm (ML)^39^.

Here, the transport emitted BC was calculated by using Hong, et al.^39^ provided source apportionment method combined with Grange, et al.^40^ reported meteorological normalization method. Such results were also validated by the widely used transport emission inventories.

Considering the public transport of Nanjing (i.e., public bus and metro) all used electricity, therefore all transport that emitted BC came from safer private transports (i.e., taxis and private cars). Then, the shifts between public and private transport could be discussed. In this work, we fully discussed the potential mechanism behind the variation of private and public transport. Our results highlighted the impacts of the restriction policy, and the higher infectivity affected the willingness of individuals on public transportation.

## Material and methods

### Study area

Nanjing, as one of the crucial cities in the Yangtze River Delta (YRD) region, East China, has subway operating mileage and passenger volume second only to Shanghai in the YRD region. In Jan 2020, Jul 2021, and 2022, Nanjing intermittently experienced complete outbreaks of SARS-CoV-2 Alpha, Delta, and Omicron, respectively. Besides, Nanjing also experienced the lockdown impacts after the lockdown of Shanghai in Apr 2022 because of the integrated transportation system of the YRD region. Therefore, Nanjing, as the only city that experienced the impacts of whole outbreaks of SARS-CoV-2, could help us better understand the relationship between SARS-CoV-2 and the impacts of human behaviours.

### Data collection

#### SARS-CoV-2 positive cases

**Figure 1:**
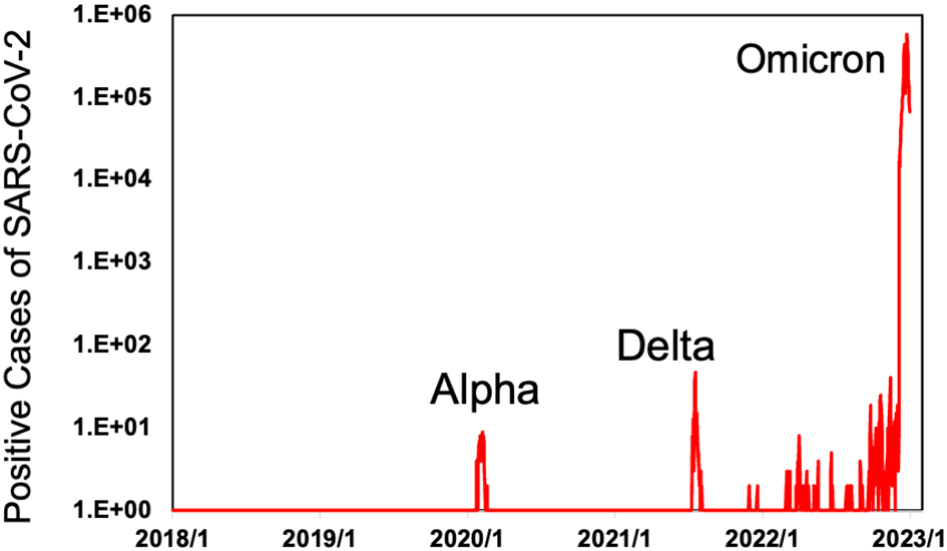
The daily number of detached SARS-CoV-2 positive cases in Nanjing.

The daily detached SARS-CoV-2 positive cases before 10th December 2022 were collected from the Nanjing Health Committee (NHC), while those after 10th December 2022 were collected from the Aliyun database. The detailed information on SARS-CoV-2 positive cases in Nanjing is shown as follows in Fig. 1.

#### Urban traffic volume

In this research, only the public buses and metros were considered as public transportation in Nanjing (public ferries, public taxis, public bicycles, and high-speed railways were omitted) and the data was collected from the Nanjing Transport website. Need to mention that the data for 2018 were calculated by the variation rate and reported data in 2019.

#### Atmospheric pollutants and pollutants emission inventory

The concentration of compounds inside fine particle matter (PM_2.5_), including black carbon (BC), nitrate aerosols (NO_3_^−^), and sulfate aerosols (SO_4_^2^), in Nanjing, were collected from the tracing air pollution in China (TAP), and the details of this database could be found elsewhere^41–43^.

The emission inventory of BC in this research came from three widely used emission inventories: the hemispheric transport of air pollution (HTAP) emission database, where details could be found elsewhere^44,45^; the multi-resolution emission inventory model for climate and air pollution research (MEIC) database, where details could be found elsewhere^46–48^; the Peking University fuel (PKU) emission database, where details could be found elsewhere^49–51^.

In this research, the transport emitted BC was collected from road transport and other ground transport those two emission sectors in the HTAP emission inventory, and the transportation emission sector in the MEIC and PKU inventories. The total emitted BC was collected from the emitted BC from the sum of whole emission sectors, which was eleven (i.e., energy, industry, fugitive, road transport, other ground transport, residential, domestic aviation, brake and tyre wear, domestic shipping, waste, and agricultural waste burning) in the HTAP emission inventory and six (i.e., power, industry, residential, transportation, agriculture, and biomass burning) in the MEIC and PKU emission inventories. The spatial resolution of these three emission inventories was 0.1° × 0.1°. Need to mention that the HTAP database only provided 2000 to 2018 emission data while PKU and MEIC databases only provided 2008 to 2017 emission data.

Therefore, the emission data used in this work was collected from the 2018 emission data in HTAP while the 2017 emission data was collected in MEIC and PKU. These three emission inventory files were cut by the Nanjing shape file combining the rgdal and raster packages in the R programming language, and the detailed information could be found elsewhere^52^.

#### Meteorological parameters

Meteorological parameters [include the 10 m u/v-component of wind (marked as U10 and V10), 2 m dewpoint temperature (D2M), 2 m temperature (T2M), boundary layer height (BLH), mean boundary layer dissipation (MBLD), surface pressure (SP), and total precipitation (TP)] were obtained from the ERA5 reanalysis dataset from the European centre for medium-range weather forecasts (ECMWF). The 0.25° × 0.25° meteorological parameters with longitude and latitude were cut by the Nanjing shape file combining the rgdal and raster packages in the R programming language.

### Linking the atmospheric black carbon concentration with urban traffic volume

#### Removing the impacts from the meteorological conditions

Here, three random forest (RF) models were built to decipher the complex non-linear function relation using the ranger package in the R programming language^53^ for weather normalization and source apportionment^39,40^. Random forest is the suitable algorithm for conducting small dataset and has been widely used in source apportionment research previously^43,54^. Previous research indicated that anthropogenic emissions (e.g., transport and factory emissions), biomass burning emissions, and meteorological conditions are the three crucial factors of variation of BC in the atmosphere^55^. Then, the concentration of BC in the atmosphere could be divided as:

1 $${\text{BC}} = {\text{BC}}_{{{\text{Biomass}}}} + {\text{BC}}_{{{\text{Factory}}}} + {\text{BC}}_{{{\text{Transport}}}} + {\text{BC}}_{{{\text{Meteorology}}}} + \varepsilon ,$$

where the biomass burning, factory, and transport emissions could increase the BC concentration while meteorological conditions could both increase (i.e., lower wind speed and boundary layer height could enhance the accumulation of pollutants) and decrease (i.e., higher wind speed and boundary layer height could dilute the pollutants) the BC concentration in the ambient atmosphere.

Recent research indicated that biomass burning could be indirectly affected by the meteorological parameters by influencing the active choices of farmers^56^. Therefore, removing the potential impacts of meteorological conditions could also remove the potential impacts of biomass burning. Here, the function relation between emission and meteorological condition with pollutant concentration (e.g., BC, NO_3_^−^, and SO_4_^2−^) could be built using the RF algorithm as follows:

2 $${\text{Pollutant}} = f_{1} ({\text{Year}}, \;{\text{Month,}} \;{\text{Whole}}, \;{\text{Meteorology}}) + \varepsilon ,$$

where Year, Month, and Whole were used to represent the local emission, Meteorology represents the meteorological parameters used here, and *f*_1_ represents the non-linear function relation learned by the RF model. Then, the normalized concentration of BC, NO_3_^−^, and SO_4_^2−^ could be calculated by this model through the method provided by Grange, et al.^40^.

#### Source apportionment of factory and transport emissions

In the previous section, the emission of BC, NO_3_^−^, and SO_4_^2−^ were calculated by the RF-based meteorological normalization functions provided by Grange, et al.^40^. The meteorological normalized concentration of aerosol particles could be seen as the emission of its precursors including BC, NO_x_, and SO_2_. After that, the concentration of BC _Normalized_ in the atmosphere could be divided into the following two sources:

3 $${\text{BC}}_{{{\text{Normalized}}}} = {\text{BC}}_{{{\text{Factory}}}} + {\text{BC}}_{{{\text{Transport}}}} + \varepsilon .$$

**Figure 2:**
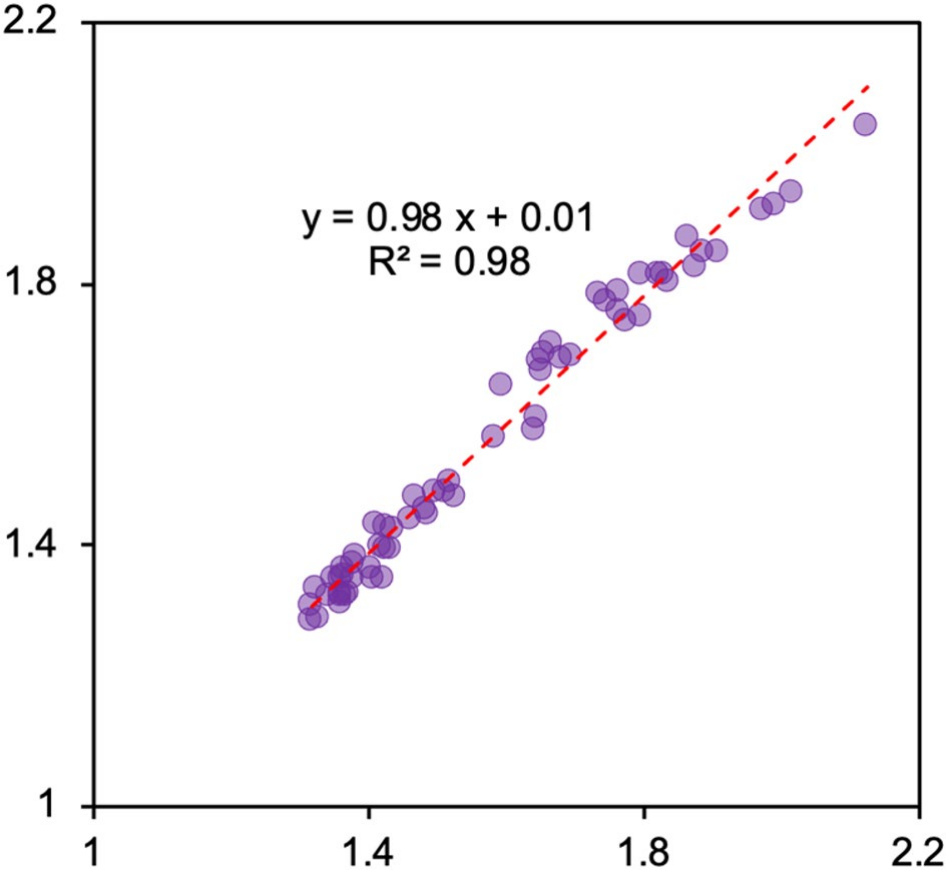
The predicted concentration of black carbon (BC) vs. the observed concentration of BC in the decomposition method by the concentration of nitrate and sulfate aerosols.

Here, using the emission of NO_x_ and SO_2_, the concentration of human activities emitted BC could be further divided into transport (e.g., gasoline exhaust emission) and factory (e.g., waste incineration, thermal power generation, and industrial emission) emissions. After that, an attribution technique was used to decompose the normalized BC (BC emission) concentration to traffic and public emitted BC by assuming RF algorithem well learned the relationship shown in Eq. (3)^39,54^. The predicted BC shows a good correlation with observed BC (Fig. 2), indicating the ML-based attribution technique could decipher the relationship between the meteorological normalized concentration of BC, SO_4_^2^, and NO_3_^−^. The attribution results were further tested with emission inventories (i.e., MEIC, PKU-Fuel, and HTAP, see Text S2 in the supplementary information). Since all public buses and metro use electricity in Nanjing, the BC emission of traffic could be explained as the usage of private transportation if neglect the emission from trucks.

## Linking the decreasing usage of public transport with health risks and economic loss

Although workers from the job sectors which could work remotely and have a positive view of working from home (WFH) experience^57^, they all showed a higher probability of returning to their workplaces after WFH during the pandemic based on the survey research in the USA^58^. Besides, since only ~ 15% of working people's job sectors were education, information technology, administrative/administrative support, and scientific research those flexible work, mainly citizens in Nanjing cannot achieve flexible work time and would not be significantly affected by telecommuting during the COVID-19 outbreak period^59,60^. Moreover, the daily traffic volume of urban transportation would not significantly shift without a significant change in the governmental restriction policy (see Table 1). Hence, the changes of commuting function in Nanjing during the pandemic periods could be explained as the impacts of policy. Therefore, although some research in the USA explained the considerable (~ 68%) decrease in the usage of public transport during and after the first outbreaks of COVID-19 as the increasing usage of WFH^61^, in China, this decrease in public transport could only be explained by the increasing health and economic risks.

**Table 1:** The daily variation of metro passenger volume in Nanjing (Unit: million people).

	Average	STD	Week1	Week2	Week3	Week4	Week5	Week6	Week7	Week8
Sunday	1.15	0.33	–	1.00	1.26	1.62	0.93	0.55	1.28	1.43
Monday	1.02	0.33	–	0.86	0.96	1.57	0.65	0.60	1.12	1.34
Tuesday	1.54	0.55	–	1.75	2.05	2.38	0.97	1.03	1.07	—
Wednesday	1.62	0.52	–	1.79	2.10	2.23	0.78	1.11	1.70	–
Thursday	1.63	0.52	–	1.90	2.12	2.09	0.68	1.22	1.74	–
Friday	1.60	0.53	–	1.97	2.14	1.90	0.62	1.22	1.77	–
Saturday	1.71	0.51	1.81	2.16	2.33	1.72	0.67	1.40	1.90	–
Workday	1.47	0.55								
Weekend	1.43	0.51								

*Note* workday and weekend showed no significant difference (p > 0.05).

During the quarantine period, people need to undertake the loss caused by failure to work correctly and the quarantine fees. Such a governmental quarantine period emphasized the risk of transmission and fatality rate and could cause a significant loss in their wage and deposits, and then, reduced the mobility of public transport^62^. Since private cars or online car-hailing could significantly decrease the potential possibility of being quarantined, it can be foreseen that people would tend to choose more private commuting during the pandemic period. Then, the following ML model could be established to predict the BC emitted by private transport (BC_Private_):

4 $${\text{BC}}_{{{\text{Private}}}} = f_{3} \;({\text{Met}},\;{\text{Period}},\; {\text{Transmission}}, \;{\text{Fatality}}, \;{\text{Case}}),$$

where Met represents the weather conditions, which could affect the consuming time of transportation (i.e., precipitation) or comfort level by affecting the sensible temperature (i.e., dew point temperature, air temperature, and wind speed); Period represents the day or not a legal vacation; The rate of transmission and fatality were used to roughly reflect the variation ([Transmission, Fatality]) of SARS-CoV-2 itself during Alpha (1, 3), Delta (2, 2), and Omicron (1, 3); where Case represents the reported SARS-CoV-2 positive patient in Nanjing.

## Results

### Variation of urban traffic volume before, during, and after the COVID-19 pandemic

**Figure 3:**
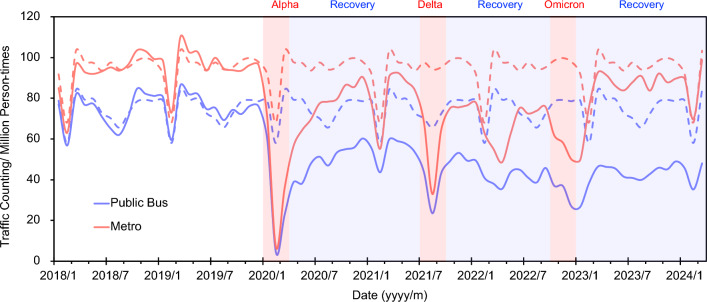
The monthly traffic counting of the public bus and metro in Nanjing, China from Jan-2018 to Mar-2024. The dot line represents the baseline of the normal behaviour calculated by the average value of each variable in 2018 and 2019 (without COVID-19 impacts).

As shown in Fig. 3 and Fig. S7, compared with the baseline distribution of transportation, public buses, metro, and taxis all showed a significant decrease after the outbreaks of SARS-CoV-2 Alpha, Delta, and Omicron. To be specific, public buses decreased by ~ 30%, ~ 37%, and ~ 66%, metro decreased by ~ 16%, ~ 30%, and ~ 49%, and taxis decreased by ~ 47%, ~ 48%, and ~ 49% in SARS-CoV-2 Alpha, Delta, and Omicron outbreaks, respectively (see Fig. 3). The difference between public (i.e., bus and metro) and private (i.e., taxi) transport reflected the different performances of citizens during the COVID-19 pandemic. Compared with the traffic volume of taxis in 2018, taxi usage decreased by ~ 6% in 2019, highlighting the potential usage of online car-hailing.

The traffic counting of public buses and metro all showed a rising tendency after the optimization of the COVID-19 response in the transportation sector, although still lower than the baseline. The decreasing usage of public buses and metro could be attributed to the decreasing demand and the development of alternative transportation. Previously, Eliasson^14^ reported that the commuting and travelling function could be significantly affected by the experiences, developments, and habits gained from the COVID-19 pandemic. Here, the decrease in traffic volume in public buses and metro during the recovery period could be seen as affected by the restrictions and recommendations to keep social distance in the case of being quarantined for 14 days, which could significantly increase the commuting cost (see details in Text S3). In contrast, the metro showed a significant decrease after the first outbreak of SARS-CoV-2 Alpha (90%), Delta (16%), and Omicron (30%), where the decreasing trend of metro during Delta and Omicron was much lower than Alpha. After the pandemic, the usage of the metro was back to 92% of the baseline while public buses are still at a stable lower level (58%). Such a results was similar with previous report in USA, in which suggest that transit patronage is likely to remain depressed by about 30% for the foreseeable future^63^.

During the SARS-CoV-2 Alpha outbreak, 93 people were caught by COVID-19 in Nanjing (Fig. 1). In order to stop the virus from spreading further, the Nanjing government closed all public places and started quarantining the people who had visited history of the outbreak areas from 12th Feb to 8th May 2020 (see Supplementary Information Text S3). Since the government was not forced to close public transport, the transport volume could reflect the decision choice of individuals during the SARS-CoV-2 Alpha outbreak period. Such regulations made the transport usage of the metro back to 48–77% of the normal usage compared to 2018 and 2019 until the SARS-CoV-2 Delta outbreaks happened in Lukou Distinct. Since Lukou is almost the southernmost part of Nanjing and far away from the downtown, such outbreaks also haven't caused the whole city lockdown. Although the city haven't been lockdown, the usage of metro only back to 36–70% of the normal usage compared to 2018 and 2019. During the following off-and-on outbreaks, the Nanjing government followed similar restrictions until the fully deregulated control in Dec 2022 (see Texts S3–S4).

### The differences in the passenger volume of public transport between workdays and weekends

To better link the relationship between the daily and monthly passenger volume of public transport in Nanjing, we collected 52 daily data on metro passenger volume from 25th Nov 2022 to 8th Jan 2023 to estimate the difference between workdays and weekends. As shown in Table 1, workday and weekend show a similar value with no significant difference (p > 0.05). Therefore, the further monthly passenger volume could somewhat represent the daily passenger volume. Besides, to obtain more general results, the day number of a month was selected as 30 in the following estimation.

### The variation of observed and normalized black carbon concentration

**Figure 4:**
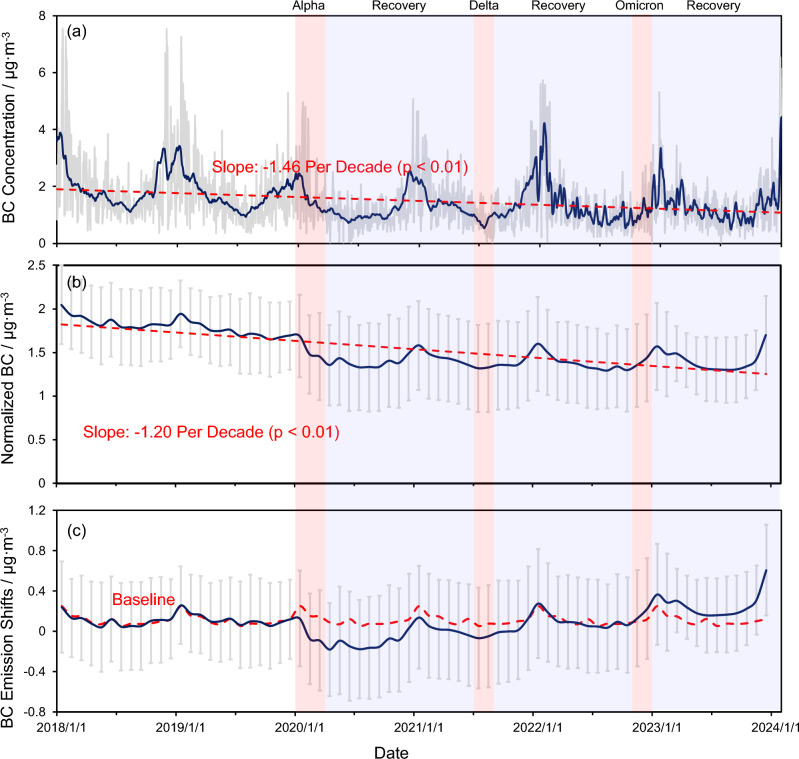
(**a**) The concentration of daily BC concentration (grey) and weekly BC concentration (navy-blue) in Nanjing, China; the red line was the trendline of the weekly BC concentration calculated by the linear regression. (**b**) The monthly BC concentration after the meteorological normalized correction; the grey line represents the uncertainty of the normalization algorithm; the red dots line represents the tendency of the normalized BC from 2018 to 2023. (**c**) The shifts of the normalized BC concentration (navy-blue) represent the difference between normalized BC concentration and the tendency calculated by the linear regression; the red dotted line was the mean value of the normalized BC concentration in 2018 and 2019. The red shadow represents the period of SARS-CoV-2 Alpha, Delta, and Omicron outbreaks, respectively, and the blue shadow represents the recovery period with no reported native-positive patients in Nanjing, China.

The atmospheric concentration of BC showed a downward trend (− 2.56 µg m^−3^ per decade) in whole from 2018 to 2023 in Nanjing (see Fig. 4). Previous thermophotometry measured research also showed a decreasing trend of the atmospheric concentration of BC in Nanjing from 2014 to 2019 (downward trend: − 1.49 µg m^−3^ per decade)^64^. However, since the impacts of meteorological conditions, the atmospheric concentration might not show the true emission of both biomass burning and fossil fuel combusted emission. Using the RF-based weather normalized function provided by Grange, et al.^40^, the normalized concentration of BC, which could reflect the shifts of emission intensity^65^, was calculated. We further compared the normalized results with the emission inventory provided by the MEIC database (see details in Text S1). The results indicated that the normalized concentration of BC showed a slightly decreasing trend (− 1.83 µg m^−3^ per decade) than the unnormalized one, indicating that weather conditions are also the unneglectable contributors to atmospheric pollution reduction action. Such a decrease could be explained by the macro-level regulation on annual BC emissions, which includes the increasing usage of e-cars and the annual emission decrease in factories. Then, the normalized BC concentration was further minused the trend line to remove the potential impacts of such a decrease since the factory emission is usually stable at the annual level (shown in Fig. 4c). The BC emission shifts in 2018 and 2019 were further used as a baseline to help quantify the shifts in transport emissions.

As shown in Fig. 4a, the BC concentration shows a severe fluctuation after Dec-2021. Such results could be attributed to the fluctuation of meteorological conditions combined with the restricted dynamic zero-COVID policy in Nanjing. When the number of COVID-19 cases increased, the government would strengthen the control of public transportation (i.e., public buses and metro) and gathering activities (i.e., entertainment and shopping) to cut down the transmission of the SARS-CoV-2 virus. Such restrictions would dramatically decrease the emission of anthropogenic air pollutants, leading to severe fluctuations after Dec-2021.

As shown in Fig. 4b, the normalized BC concentration significantly decreased after the first outbreak of SARS-CoV-2 Alpha in Jan-2020. Such changes could be attributed to the decrease in automobile usage. At the beginning of the pandemic, the high fatality rate of SARS-CoV-2 Alpha increased the usage of remote offices and then further decreased automobile emission^59^. Besides, although experienced the other two outbreaks of SARS-CoV-2 Delta and Omicron in Jul-2021 and Dec-2022, since the decreasing toxicity of the SARS-CoV-2 virus, the emission of BC was slightly increased and overcome the baseline concentration after the outbreaks of SARS-CoV-2 Omicron and the liberalization of epidemic control in Dec-2022. Such results indicated that the experiences, developments, and habits of people, which were gained from the lockdown period^14^, seem to have disappeared with the decreasing toxicity and the continually optimized policies.

Another explanation could be the strong infectiousness of Delta and Omicron increasing the possibility of being infected. Considering the policy of the Nanjing government before Dec-2022, the longer quarantine time and higher costs could lead citizens to add the risk of being quarantined as a part of commuting, and therefore, limit the usage of public transport by citizens. Thus, more people were willing to choose some safe transportation, including private cars and online car-hailing services^66^. Such changes in daily behaviour patterns would further affect the emission pattern and finally affect the atmospheric concentration of air pollution.

## Discussion

### The variation of transports-emitted black carbon

As shown in Fig. 4, the public transport emitted BC in 2018 and 2019 was more stable than during the pandemic period. Before the COVID-19 pandemic, the emission of BC would increase before the vacation of Chinese New Year and decrease during and after the vacation (from February to May). After the outbreaks of SARS-CoV-2 Alpha happened in January 2020, the private transports emitted BC concentration decreased to 0.35 µg m^−3^ while the baseline was ~ 0.49 µg m^−3^ before the pandemic. Besides, the decrease in BC emissions from private transports, which usually ended in July (the beginning of summer vacation) previously, was lasting to September 2020. Moreover, even in the recovery period, where China was not affected by the coronavirus pandemic, the emission pattern still showed a little bit differently than before. The peak and inflexion point of private transports emitted BC came one month earlier than the baseline (2018 to 2019). These abnormal emission patterns could be seen as evidence of how the pandemic affects human behaviours on transportation.

The most interesting thing was the reflection of both private and public transport emissions during the outbreaks of the SARS-CoV-2 Delta in Lukou, Nanjing. The public transport emissions significantly decreased while the emission of private transport increased. Considering the fatality rate of SARS-CoV-2 Delta was greatly lower than Alpha^9^, this variation could be attributed to the tough quarantine policy, where the person with a spatial–temporal overlap with a positive patient needs healthy isolation for 14 days (see details in Text S3). Such a policy might increase the cost of public transportation (this part will be fully developed in the next section) and then increase the use of private transport (i.e., private vehicles, online car-hailing, and motorcycles). This policy caused a dramatic increase in private transport emissions in August, which was 2.2 times higher than the baseline (0.05 µg m^−3^).

### The correlation of public and private transport-emitted black carbon

**Figure 5:**
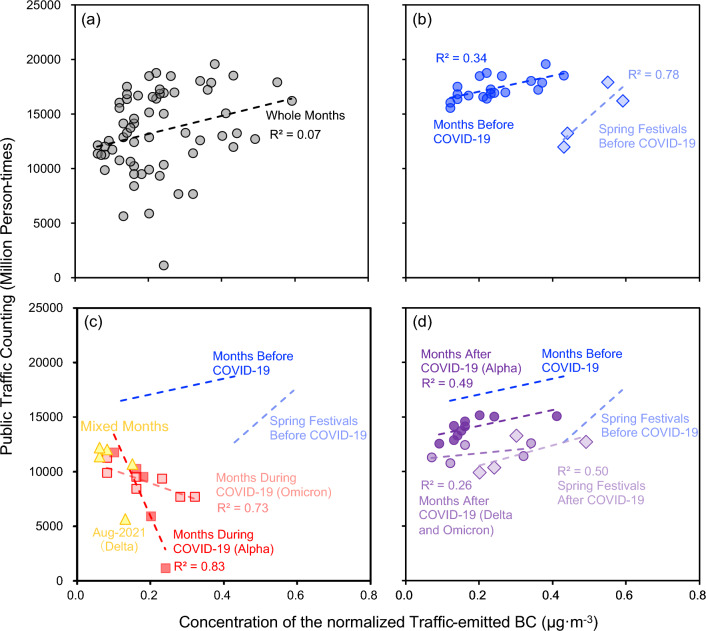
The dots plot of monthly traffic-emitted black carbon (BC) in Nanjing and the monthly traffic counting of public transport (including public bus and metro) from January 2018 to February 2023.

As shown in Fig. 5, the private transport emissions showed a positive correlation (slope = 0.03, R^2^ = 0.20, p < 0.01) with public transport emissions before the novel coronavirus pandemic, indicating the increase in public transport might accompany the increase in private transport. Such results could be attributed to the increase in the permanent population in Nanjing. Since the increasing of permanent population would not change the population structure, then, increased the usage of both private and public transport. After the pandemic, the correlation between public transport and private transport almost showed a flat relationship, indicating the use of public transport did not increase as the increase of private transport due to the deficient of comfort, safety, and reliability^67^. This part could represent the relationship between private and public transport after the impact of COVID-19 on human behaviour^20,25^. The whole effect could be divided into two parts, the pure dropping down of the usage of public transport and the decline of public transport with the increase of private transport [reflected by the increase of BC compared with the baseline in Fig. 5c]. The former decline could be attributed to the more use of telecommuting, which could directly decrease the demand for transportation^60^. The latter antagonism effect could be attributed to the demand shifts from public transport to private transport in the urban area^68,69^.

Unlike the non-pandemic period, private transport emissions showed a negative correlation (slope = − 0.17, R^2^ = 0.93, p < 0.01) with public transport emissions during the pandemic. Need to mention that the emissions in January 2020 do not show the same pattern as the other four months during the pandemic since the outbreaks of SARS-CoV-2 Alpha in Nanjing were found in February 2020 (see Text S3). However, people in Nanjing tend to use more automobiles (see the red diamond in Fig. 5c) since the tremendous media information about SARS-CoV-2 Alpha outbreaks happening in other provinces was reported. After the first positive cases in Nanjing had been announced, the usage of public transport significantly dropped while the use of automobiles increased. Such a phenomenon could be used to explain the abnormal increase in air pollutants in Beijing^70^ and Delhi^71^.

The blue diamond in Fig. 5b indicates that the Spring Festival also affected people's commuting function. Usually, the usage of public transport remaining the same level in the beginning while decreasing at the end. On the contrary, the usage of private transport considerably increases in the beginning while decreasing to the same level at the end of the vacation. Such results might somehow reflect the demand shifts of citizens in the urban area. Such demands could also be affected by the COVID-19 pandemic. The slope between private and public transport after the pandemic showed a decrease compared with the slope before COVID-19 (Fig. 5d). Such results also reflected that more people turned to using private transport after the pandemic.

From SARS-CoV-2 Alpha to Delta and Omicron, as shown in Fig. 5c, with the decrease in the fatality rate of viruses, the red point moves along the red line to the upper left corner (more public transports were used). However, since the Nanjing government started to take control of anthropogenic activities after January 23, 2020 (see Text S3), the emissions started to show an abnormal pattern of other points before the pandemic (see blue points in Fig. 5). Moreover, since no positive cases were found in Nanjing in January 2020, public transportation was not dropped into the line of emission during the pandemic. After the first announcement of SARS-CoV-2 Alpha cases in Nanjing in February, public and private transport soon dropped to the red point with the lowest public transport. This is a case of how the fatality rate of SARS-CoV-2 affects people's commuting decisions. Another interesting thing was the slope between private and public transport before and after COVID-19 was almost the same and only the intercept was changed (Fig. 5d).

The prevention and control measures in Nanjing became strict in late November (see Text S3), giving another case showing how governmental policy affects people's commuting decisions [see Fig. 5(d)]. From October to December 2020, even though no outbreaks of COVID-19 were announced in Nanjing, the normal prevention and control measures caused a significant increase (R^2^ = 0.92, p < 0.01) in automobile usage.

### The transmission and fatality of virus affected the variation of urban transportation

**Figure 6:**
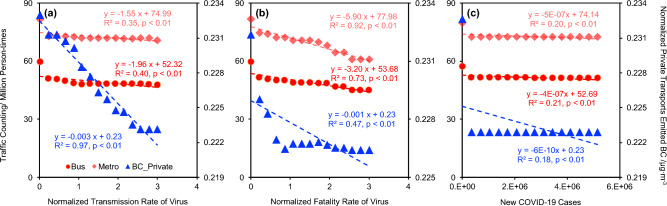
The partial dependency results of the normalized transmission rate (**a**), normalized fatality rate (**b**), and new COVID-19 cases (**c**) to public bus, metro, and private transport emitted BC.

In Section "The correlation of public and private transport-emitted black carbon", we show the correlation between public and private transport before, during, and after the COVID-19 pandemic. Such results indicated that both private and public transport could be affected by the COVID-19 pandemic, where the impact of private transport is usually lower than public transport since it has a lower infection (or quarantined) risk compared with public transport. Considering the policy of local government usually affected by the transmission and fatality rate of viruses and the number of positive patients, an ML-based model (see function F6) was built to decipher the relationship between virus and transportation volume using the monthly panel data. The partial dependency results between the transmission and fatality rate of viruses and the number of positive patients of the virus are shown in Fig. 6.

All transportation, including public bus, metro, and private transport, were significantly (p < 0.01) affected by the transmission and fatality rate of SARS-CoV-2 and the number of positive individuals (Fig. 6), which was similar to the results in previous research^1,2,59^. However, the partial dependency results between new COVID-19 cases and traffic volume of the public buses, metros, and private transport were not different much after the outbreaks of the SARS-CoV-2 virus. Similarly, the traffic volume of private transports did not decrease much during the SARS-CoV-2 Alpha (normalized fatality rate equals 3) and Delta (normalized fatality rate equals 2).

## Conclusion

Our results indicated that the SARS-CoV-2 outbreaks only limited transport in urban areas with a higher fatality and lower transmission abilities (SARS-CoV-2 Alpha). With the decrease in fatality rate as well as the increase in transmission abilities, private transport would significantly increase.

This research provided a method to use the atmospheric concentration of BC aerosols to reflect the variation of both public and private traffic in the target city using a weather-normalized correction combined with the source apportionment method.

Our method could be used in other cities when the direct panel dataset is hard to collect or achieve. With the widely observed dataset of BC and the worldwide reanalysis of meteorological parameters, it was hopeful to conduct global wide research to compare the spatiotemporal distribution of traffic activities.

With the latest updated data (until April 2024), both private (115%) and metro (92%) increased significantly compared to the baseline after COVID-19, while public buses are still at a similar level (~ 50%) to the last three years. The decline of public buses was accelerated by the pandemic and can be expected to continue in the following years, indicating that future public transport management should focus more on the public metro.

### Supplementary Information

Supplementary Information.

## Data Availability

The datasets used and analysed during the current study available from the corresponding author on reasonable request.
