# Supplementary material for: The effect of quarantine policy on pollution emission and the usage of private transportation in urban areas
Source: Sci Rep. 2024 Jul 8;14:15752. doi: 10.1038/s41598-024-66685-8 (PMC11231271; doi:10.1038/s41598-024-66685-8)
Supplement: Supplementary file 1 — Supplementary Information. [file 41598_2024_66685_MOESM1_ESM.docx]

**Supplementary Information**

*for*

**The effect of quarantine policy on pollution emission and the usage of private transportation in urban areas**

Yihang Hong^1,2^, Ke Lu^1,*^

*^1^ School of Management Science and Engineering, Nanjing University of Information Science & Technology, Nanjing 210044, China*

*^2^ Department of Economics, University of Reading, White Knight RG66UR, United Kingdom*

**^*^Corresponding author:** Ke Lu (k.lu@nuist.edu.cn)

# Text S1. The Factory and Traffic Emitted BC from the Emission Inventory

In this research, three widely used emission inventories were used to prove the reliability of the decomposed results. The three emission inventories were: The multi-resolution Emission Inventory model for Climate and air pollution research (MEIC), Peking University fuel (PKU), and hemispheric transport of air pollution (HTAP) emission inventories. Firstly, the reliability of these three emission inventories was tested throw the correlation between the factory and transport emitted black carbon (BC) within these inventories. Although the factory emitted BC from HATP showed a large variance from the emission from MEIC and PKU inventories, they still exhibited a similar trend to some extent. As shown in Figure S1, all emission inventories show a significant (p < 0.01) positive correlation, especially the relationship between PKU and MEIC emission inventories (R^2^ = 1, n = 12). Such results indicated that the emission inventory could well reflect the monthly emission shifts, and the differences mainly came from the calculation function.


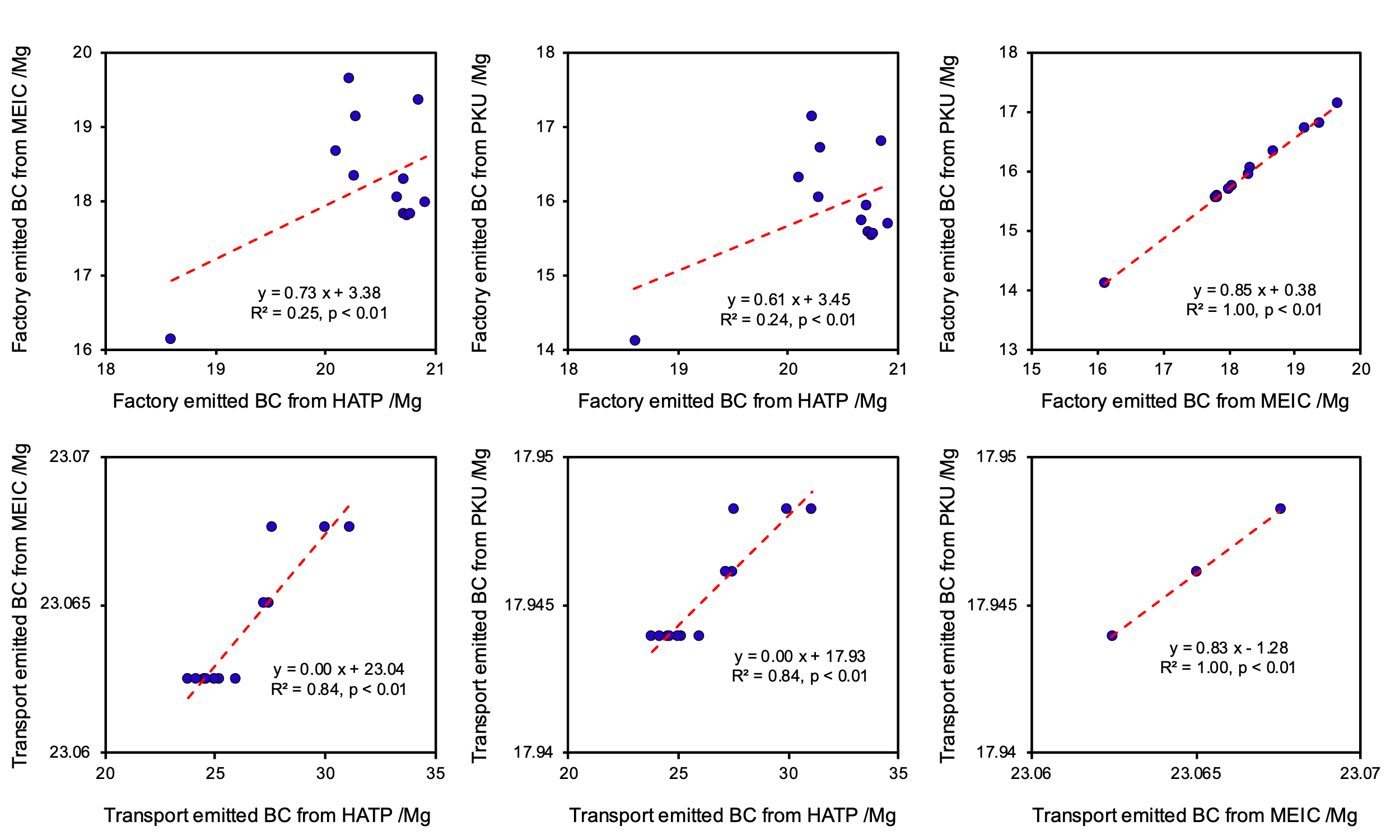


**Figure S1.** The correlation between factory and transport emitted BC from the MEIC, PKU, and HATP emission inventory.

Except for the BC concentration that was normalized by the meteorological normalization, we also estimate the normalized sulphate (SO_4_^2-^) and nitrate (NO_3_^-^) concentrations. Since the precursor of SO_4_^2-^ (sulphur dioxide, SO_2_) and NO_3_^-^ (nitrogen oxides, NO*_x_*) were mainly emitted by factories and vehicles, respectively, then, the normalized SO_4_^2-^ and NO_3_^-^ could represent the factory and transport emissions to some extent. As shown in Figure S2, although still containing some noises, the significant positive correlation (Slope > 0, p < 0.01) indicated that the normalized results have already captured some features of target sources. These normalized SO_4_^2-^ and NO_3_^-^ concentrations were further used as the input variables to decompose the normalized BC concentration into transport-derived and factory-derived BC concentrations (see details in Section “Atmospheric pollutants and pollutants emission inventory”).


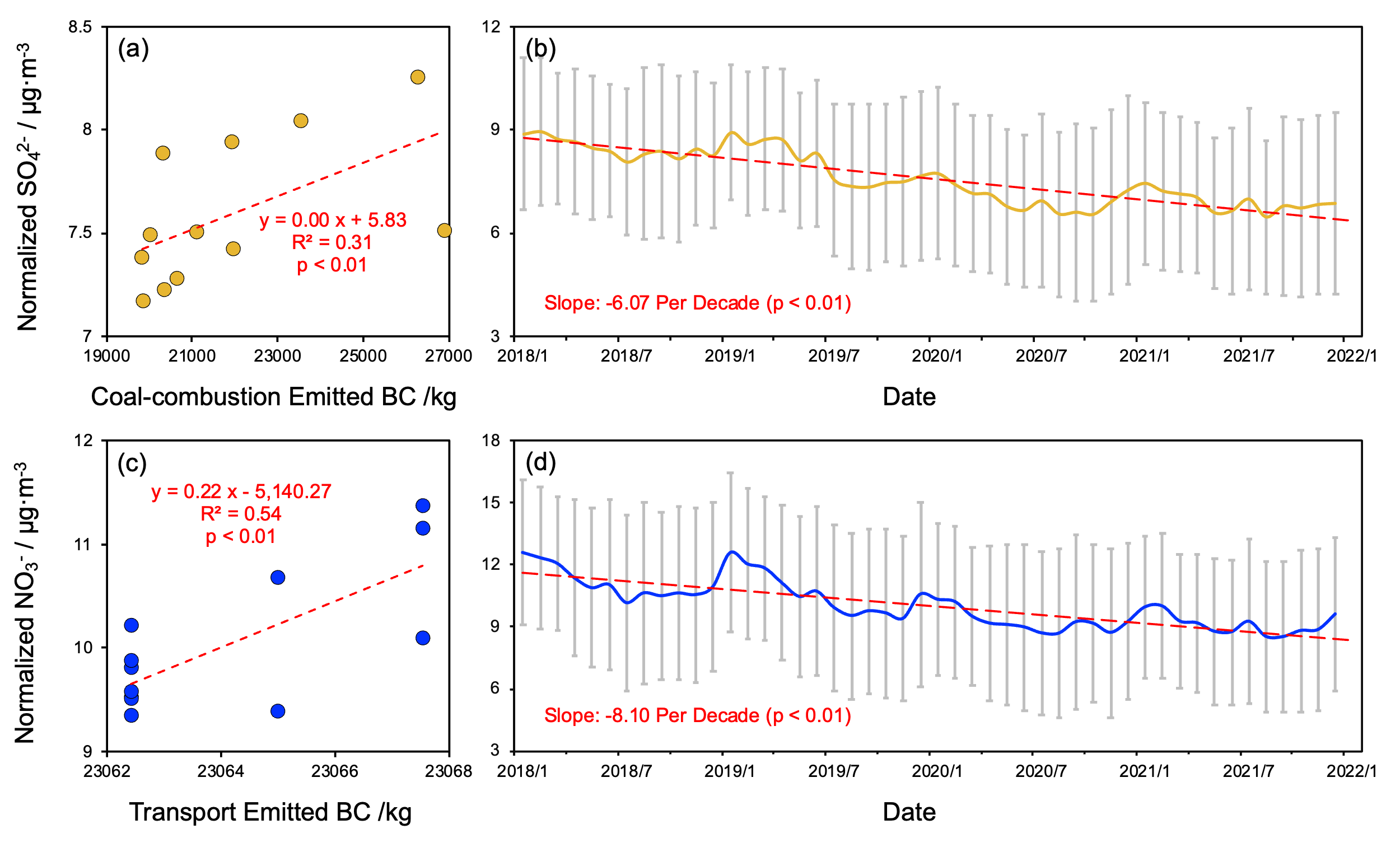


**Figure S2.** (**a**) The correlation between coal-combustion emitted BC (including the sources of industry, power, and residential) from the MEIC emission inventory and normalized concentration of SO_4_^2-^; (**b**) The monthly variation of SO_4_^2-^ with the uncertainty (grey line) and trend (red dots line). (**c**) The correlation between transport emitted BC and normalized concentration of NO_3_^-^; (**d**) The monthly variation of NO_3_^-^ with the uncertainty (grey line) and trend (red dots line).

# Text S2. **Compared the Normalized Concentration with Emission Inventory**

Previously, Grange *et al.* ^1^ provided a meteorological normalization method driven by the Random Forest (RF) algorithm 1. Such a method could remove the impacts from weather conditions, and the normalized concentration of target pollutants could reflect the shifts of emission intensity ^2^. Here, using the three emission inventories mentioned above, we further test the reliability of the BC concentration with meteorological normalization by estimating the correlation between the whole emitted BC with the BC concentration with meteorological normalization. Besides, to prove the reliability of the decomposed transport-derived BC concentration with meteorological normalization.

As shown in Figure S3, the normalized BC concentration showed a significant positive correlation (Slope > 0, p < 0.01) with total sector BC emission from HATP (R^2^ = 0.66), MEIC (R^2^ = 0.57), and PKU (R^2^ = 0.64) emission inventories, proving the reliability of the meteorological normalization method provided by Grange *et al.* ^1^.


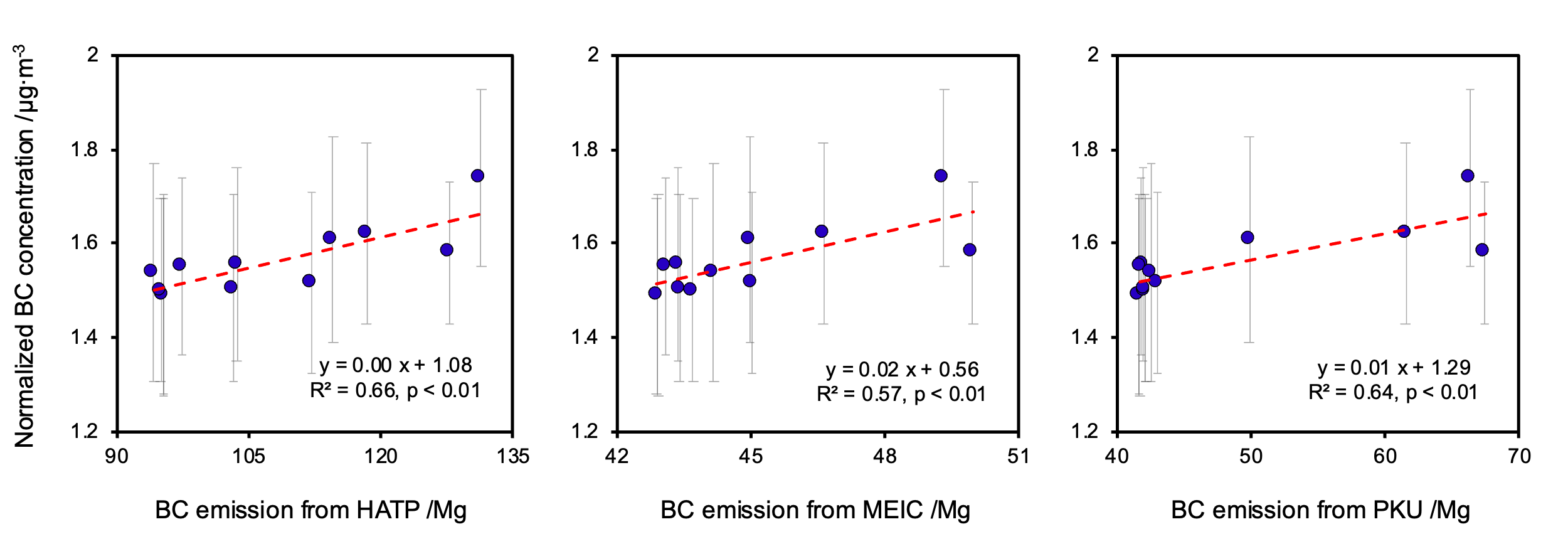


**Figure S3.** The correlation between BC emission from the HATP, MEIC, and PKU emission inventories and normalized BC concentration.

Then, we further compared the decomposed transport-derived BC concentration with the transport-emitted BC from the emission inventories mentioned above. The decomposed transport-derived BC concentration showed a significant positive correlation (Slope > 0, p < 0.01) with HATP, MEIC, and PKU emission inventories (see Figure S4). Such results indicated that the decomposed results were trustable and proved that further discussion in this research was reliable. Besides, the decomposed transport emitted BC concentration during the COVID-19 period was slightly lower than the BC concentration before the COVID-19 period, indicating the lower usage of vehicles after the SARS-CoV-2 Alpha outbreaks in Jan-2020.


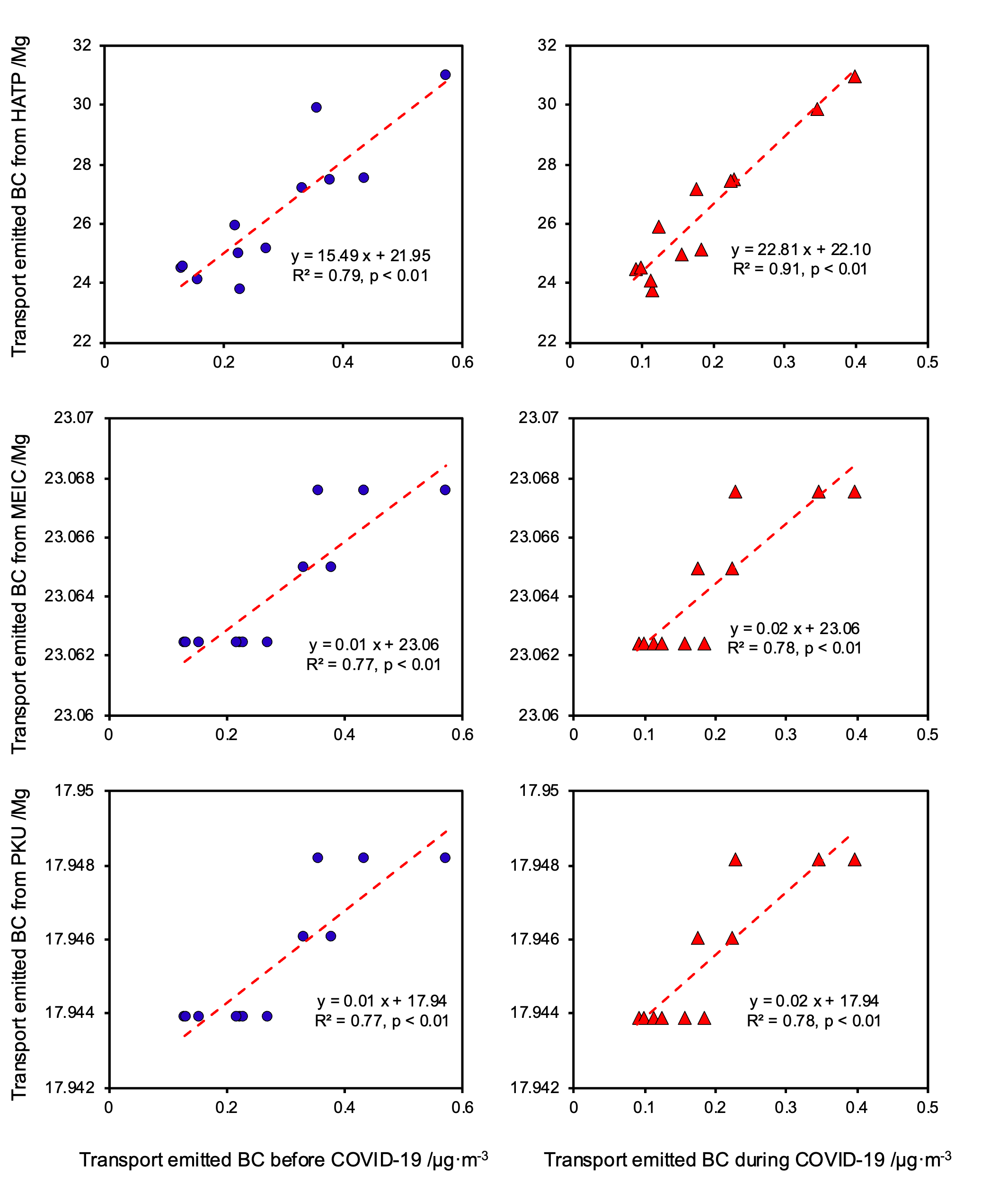


**Figure S4.** The correlation between transport emitted BC from the MEIC emission inventory and normalized transport BC from our decomposition results.


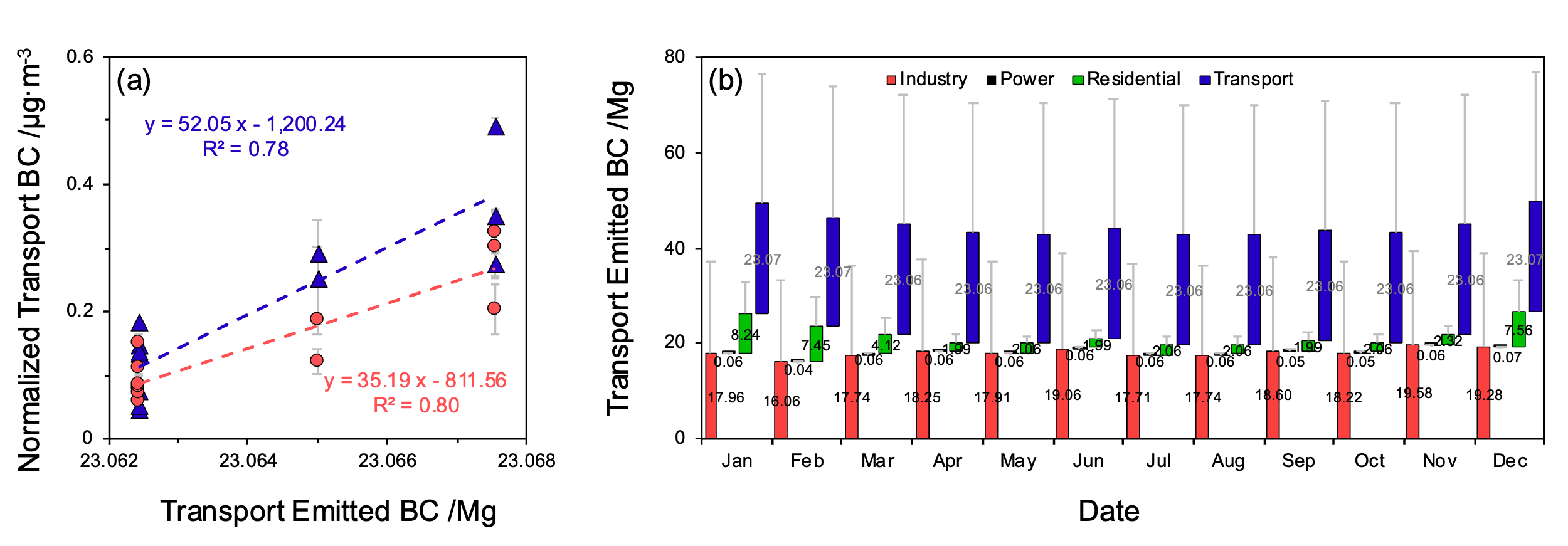
**Figure S5.** (**a**) The correlation between transport emitted BC from the MEIC emission inventory and normalized transport BC from our decomposition results. (**b**) The variation of anthropogenic emitted BC aerosols in Nanjing from the MEIC emission inventory.

# Text S3. **The Detailed Information of Notice During COVID-19 in Nanjing**

The detailed information on the notice file during the outbreaks of SARS-CoV-2 in Nanjing could be found on Nanjing Health Committee (NHC) website^[[1]](#footnote-1)^. In this notice, the Nanjing government suggested that the people outside the city postpone coming to Nanjing during the epidemic prevention and control period (beginning on 12th Feb 2020). People with signs of fever or cough need to quarantine for 14 days, and people who came from COVID-19 outbreak areas or contacted people from COVID-19 outbreak areas need to quarantine for 14 days. As the SARS-CoV-2 Alpha receded in China, local governments are implementing measures to stimulate consumption and facilitate the return to normal life and business operations after May 8th, 2020^[[2]](#footnote-2)^.

The detailed information of the notice file during the outbreaks of SARS-CoV-2 Delta in Nanjing could be found elsewhere^[[3]](#footnote-3)^. In this notice, the Nanjing government suggested that the person who wants to leave Nanjing needs a negative nucleic acid test within 48 hours; people outside Nanjing have been advised to postpone their travel plan during the epidemic prevention and control period (beginning on 21st July 2021); the enclosed public space in Nanjing has also been closed since 21st July 2021; only Lukou Street has been lockdown after these outbreaks.

# Text S4. **The Detailed Information of the “10 New Measures” Guideline**

On 8th Sep 2022, the Chinese central government published the “10 new measures” to optimize the COVID-19 response. Detailed information on this “10 new measures” guideline could be found on the website of the Chinese central government^[[4]](#footnote-4)^. The guideline indicated that asymptomatic or mild COVID-19 cases were suggested to stay at home until suffering breathing difficulties or other serious health issues. Besides, the guideline pointed out that homebound patients could end their quarantine period when they have no obvious symptoms and take two negative consecutive nucleic acid tests.

On 26th Dec, 2022, the National Health Commission of China issued an announcement: with the approval of the State Council, as of 8th Jan, 2023, the preventive and control measures for Class A infectious diseases under the Law of the People's Republic of China on Prevention and Treatment of Infectious Diseases related to novel coronavirus infections will be lifted. Additionally, it will no longer be managed as a quarantinable infectious disease under the Law of the People's Republic of China on Frontier Health and Quarantine.

# Text S5. **The correlation between the traffic counting of public bus and metro.**

As shown in Figure S5, the traffic counting shown no significant difference between public bus and metro, indicating that the demand of urban public transportation was usually stable even during the SARS-CoV-2 outbreaks.


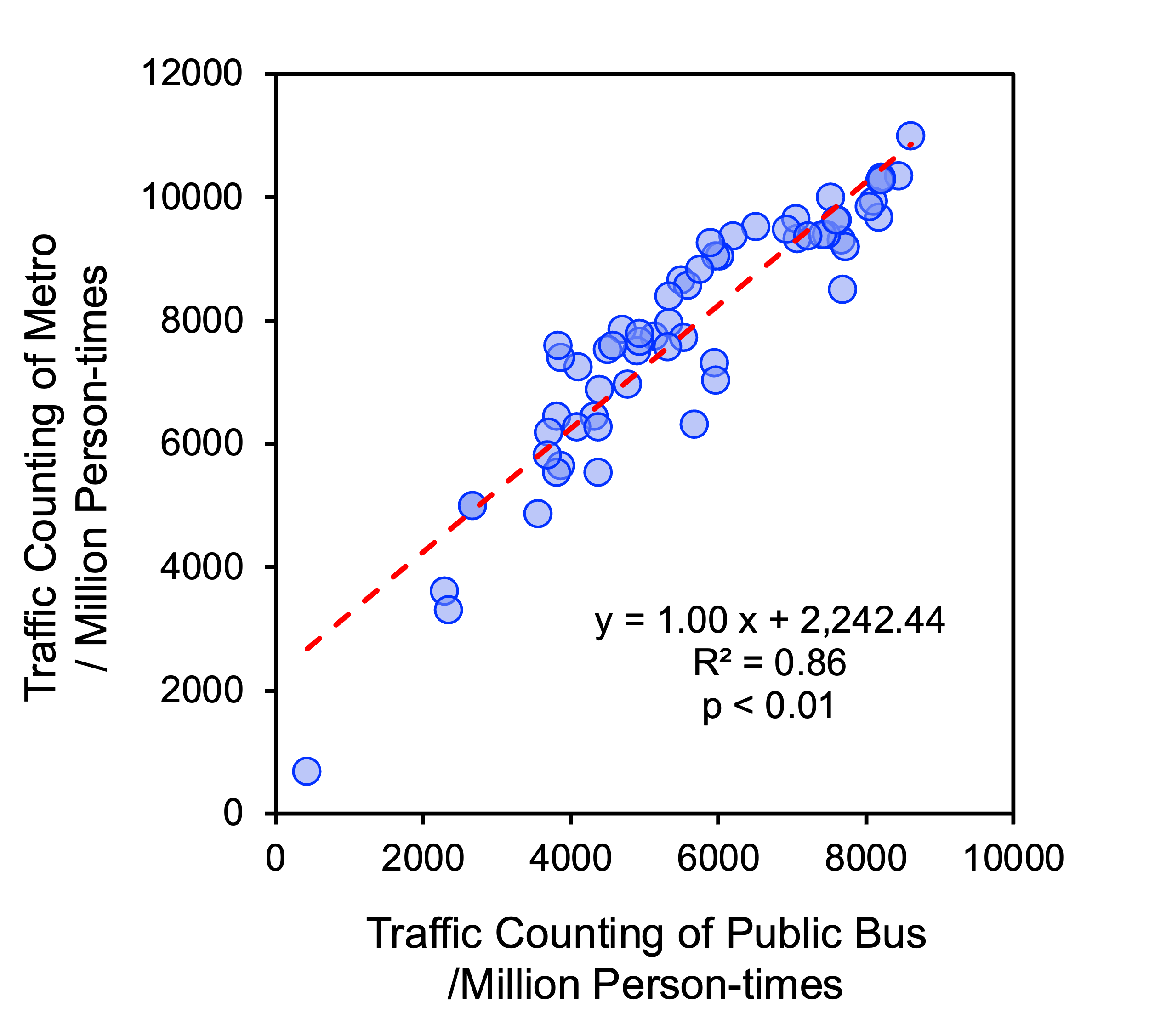


Figure S6. The corrlation between traffic counting of public bus and metro.

Uages of Taxis (million people)

Date (yyyy/m)

Figure S7. The traffic volumn of the usage of public taxis in Nanjing.

# Reference

1. Grange, S.K., Carslaw, D.C., Lewis, A.C., Boleti, E., Hueglin, C., 2018. Random forest meteorological normalisation models for Swiss PM10 trend analysis. Atmospheric Chemistry and Physics 18, 6223-6239.

2. Grange, S.K., Carslaw, D.C., 2019. Using meteorological normalisation to detect interventions in air quality time series. Science of The Total Environment 653, 578-588.

1. Website: https://wjw.nanjing.gov.cn/njswshjhsywyh/202002/t20200213_1790857; Last access: 8^th^ April 2023. [↑](#footnote-ref-1)
2. Website: https://www.gov.cn/xinwen/2020-05/08/content_5509978.htm; Last access: 25^th^ April 2024. [↑](#footnote-ref-2)
3. Website: https://wjw.nanjing.gov.cn/njswshjhsywyh/202107/t20210721_3080549.html; Last access: 8^th^ April 2023. [↑](#footnote-ref-3)
4. http://english.www.gov.cn/statecouncil/ministries/202212/08/content_WS6391e242c6d0a757729e4211.html; Last access: 8^th^ April 2023. [↑](#footnote-ref-4)
